# Evidence that histone H1 is dispensable for proper meiotic recombination in budding yeast

**DOI:** 10.1186/s13104-015-1246-1

**Published:** 2015-06-30

**Authors:** George S Brush

**Affiliations:** Department of Oncology, Wayne State University School of Medicine, Detroit, MI USA; Molecular Therapeutics Program, Barbara Ann Karmanos Cancer Institute, Detroit, MI USA

**Keywords:** Hho1, Meiosis, Sporulation, Genetic map distance, Crossover interference

## Abstract

**Background:**

Histone H1, referred to as the linker histone, associates with the nucleosome core particle. While there is indication that the budding yeast version of histone H1 (Hho1) contributes to regulation of chromatin structure and certain chromatin-related processes, such as DNA double-strand break repair, cells lacking Hho1 are healthy and display subtle phenotypes. A recent report has revealed that Hho1 is required for optimal sporulation. The studies described here were conducted to determine whether Hho1 influences meiotic recombination, an event that occurs during sporulation, involves generation and repair of DNA double-strand breaks, and is critical for spore viability.

**Findings:**

Through tetrad analysis, cells with or without Hho1 were compared for meiotic reciprocal recombination events within several chromosome XV intervals. Parameters investigated included crossover frequency (genetic map distance) and crossover interference. No significant differences were detected between the two cell types. In agreement with earlier studies, spore viability was not affected by Hho1 absence.

**Conclusion:**

These data suggest that complete absence of Hho1 from chromatin does not affect reciprocal recombination between homologous chromosomes during meiosis. Therefore, the basal level of Hho1 that remains after its reported depletion early in meiosis is unlikely to be important for regulating recombination. Furthermore, the subsequent accumulation of Hho1 as the haploid products mature does not appear to be crucial for spore viability.

## Background

Primary chromatin structure in eukaryotes is defined by the repeating nucleosome core particle, which consists of approximately 146 base pairs of DNA wrapped 1.65 times around a histone octamer containing two subunits each of H2A, H2B, H3, and H4 [1]. Linker DNA connects the core particles, and the full nucleosome can also include histone H1, the linker histone, which associates with the outside of the core particle structure where the DNA enters and exits [2, 3]. It is thought that H1 contributes to higher order chromatin structure by promoting proper chromatin condensation [4]. Interestingly, studies in a variety of eukaryotic cells have shown variability in the stoichiometry of H1 molecules per nucleosome, with values in wild type vertebrate cells ranging from 0.45 to as high as 1.3 (see [5]). By contrast, the core particle itself has a strictly conserved stoichiometry.

Early functional studies through in vitro strategies suggested that H1 could influence transcription. In general, H1-mediated repression of transcription was observed, but evidence of positive regulation was also reported (see [6]). With this backdrop, it was perhaps surprising when an in vivo study revealed that H1 is not essential for viability in *Tetrahymena thermophila*, and that its absence, while affecting chromatin structure, does not affect transcription on a global level [7, 8]. The situation in higher eukaryotes is more complicated given that several H1 isoforms exist. However, a triple null mouse mutant has been generated that is depleted of H1 by approximately 50% and is embryonic lethal, indicating that H1 is required for mammalian development [9]. Viable embryonic stem cells can be derived from this mouse model, and, as observed with *Tetrahymena,* chromosome structure is altered but global transcription only subtly affected in these cells [10]. Nonetheless, they are defective for differentiation [11].

The existence of H1 in the budding yeast *Saccharomyces cerevisiae* was not confirmed until the entire genome was sequenced, upon which the presence of a single gene, *HHO1*, was identified with considerable similarity to H1 genes from other species [12, 13]. Early biochemical analysis suggested a very low Hho1 stoichiometry with approximately one H1 molecule per 37 nucleosomes [14], but a subsequent study indicated approximately one H1 molecule per four nucleosomes [15]. Deletion of *HHO1* revealed that Hho1 is not required for viability [13, 16]. While initial work indicated no alteration in chromatin structure in cells lacking Hho1 [16], more recent experiments with a number of techniques have indicated that higher order chromatin structure is altered in the absence of Hho1 [17, 18]. However, as has been seen in other systems, yeast cells lacking H1 experience only subtle alterations in gene expression. In fact, one global study revealed that only 27 genes were affected 2-fold or more by Hho1 absence, and all of these genes were down-regulated in mutant *versus* wild type cells [19].

In addition to the role of Hho1 in transcription, DNA repair has been investigated. Genetic experiments showed that Hho1 presence influences DNA double-strand break repair in particular by restraining homologous recombination without affecting non-homologous end joining [15]. Recently, Hho1 behavior and function during sporulation have also been analyzed in considerable depth [20]. Sporulation is a starvation response in a yeast diploid that involves meiosis, with programmed reciprocal recombination occurring preferentially between homologous chromosomes (see [21]) during prophase I, followed by maturation of the haploid products into an ascus containing four spores. It was found that the Hho1 steady state level decreases at an early stage of sporulation, including the time when meiotic recombination would be expected to occur [20]. This decrease is suspected to be functionally linked to depletion of Ume6, a repressor of early meiotic genes [22], and could also be important for relieving inhibition of homologous recombination. At later stages of sporulation, Hho1 accumulates to a considerable extent and is involved in chromatin compaction. Cells without Hho1 show a delay in sporulation progression and a decrease in sporulation efficiency relative to wild type cells. However, data from both that study [20] and an earlier one [16] indicate that spore germination (i.e., viability) is unaffected by Hho1 status.

The work presented here was undertaken to determine whether complete absence of Hho1 could have an influence on meiotic recombination. The rationale behind this line of experimentation is that a certain threshold level of Hho1 may be required to restrain homologous recombination and thereby provide a proper balance of crossover events. To test the role of Hho1, a classical genetic approach was used to define recombination patterns in several genetic intervals.

## Methods

### Yeast strains

Parental strains EAY1108 and EAY1112 that provide multiple markers on chromosome XV for recombination analysis were kindly provided by Eric Alani (Cornell University) [23]. YGB881 (*MATα hho1Δ::kanMX4*) was generated from EAY1112 through PCR-based gene disruption [24]. Genomic DNA was isolated based on a standard method [25] from the *hho1Δ::kanMX4* strain in the *MAT***a** Yeast Knockout Collection (GE Dharmacon; [26]) and used as a template to amplify the *kanMX4* module [27] with oligodeoxynucleotide primers (IDT) designed to anneal upstream and downstream of the natural *HHO1* open reading frame:

1. 5′-CTGATAATGCTTGGCAGCGAGGG-3′ (upstream).
2. 5′-CTAATAGTGATGGGACACAAAAATGAAGAAAG-3′ (downstream).

The PCR fragment was transformed into EAY1112 by a lithium acetate procedure [28], and a recombinant (YGB881) was selected with G418 (Corning). Deletion of *HHO1* was confirmed by PCR (see “DNA and protein analyses*”* below). YGB881 was then mated to EAY1108, and haploids were generated through sporulation. The haploid strains used in this study were:

- YGB1036: *MATα, ho::hisG, lys2, ura3, leu2::hisG, trp1::hisG, URA3*-*CEN15, iLEU2*-*chXV, iLYS2*-*chXV, hho1Δ::kanMX4.*
- YGB1037: *MAT***a***, ho::hisG, lys2, ura3, leu2::hisG, trp1::hisG, ade2::hisG, his3::hisG, TRP1*-*CEN15, hho1Δ::kanMX4.*
- YGB1038: *MATα, ho::hisG, lys2, ura3, leu2::hisG, trp1::hisG, URA3*-*CEN15, iLEU2*-*chXV, iLYS2*-*chXV.*
- YGB1039: *MAT***a***, ho::hisG, lys2, ura3, leu2::hisG, trp1::hisG, ade2::hisG, his3::hisG, TRP1*-*CEN15.*

YPD (1% (w/v) yeast extract, 2% (w/v) peptone, 2% (w/v) dextrose) liquid or solid (2% (w/v) agar) media were used for routine cell growth, and SPM (1% (w/v) potassium acetate, 2% (w/v) agar) was used for sporulation. Solid synthetic complete (SC) media (see [25]) lacking individual supplements were used for scoring of markers in the tetrad analysis. All incubations were carried out at 30°C.

### DNA and protein analyses

Cells were grown from frozen stock as patches on solid YPD and then incubated in liquid YPD. For the DNA analysis, the final cultures were inoculated at a starting OD_600_ ≈ 0.5 from liquid culture and incubated overnight (20.4 h). Cells from 5 ml of cultures were harvested, genomic DNA was prepared [25], and the *HHO1* locus was analyzed by PCR using the following oligodeoxynucleotide primers (IDT):

3. 5′-AAGAGGAGGAGCAACTATAGATTTGGG-3′ (upstream).
4. 5′-GTCTCGCCGGGCTTCTACGG-3′ (downstream).

The samples were subjected to electrophoresis through a 1% (w/v) agarose gel and stained with ethidium bromide (Fisher). The expected PCR product sizes for *HHO1* and *hho1Δ::kanMX4* using these primers are 1.3 and 2.1 kilobase pairs, respectively. For protein analysis, cells were patched onto solid YPD from frozen stocks, incubated over two nights, and then used to directly inoculate liquid YPD cultures. After incubation for 26 h, 6 OD_600_ units of cells were harvested by centrifugation, washed with 1 ml cold H_2_O, and stored at −70°C. Denatured crude extracts were prepared based on a trichloroacetic acid (TCA)/bead-beating method [29]. Protein concentration was measured using the RC DC Protein Assay (Bio-Rad) with bovine serum albumin as the standard, and 30 μg of protein from each crude extract were subjected to electrophoresis through a 10% (w/v) denaturing polyacrylamide (37.5:1 acrylamide:bis-acrylamide; Fisher) gel and transferred to 0.45μ nitrocellulose (GE). The blot was first stained with 0.2% (w/v) Ponceau S (Fisher) in 3% (w/v) TCA, and then immunostained for Hho1 and tubulin. Primary antibodies used were rabbit anti-Hho1 (Abcam) and rat anti-tubulin-α (Bio-Rad). Secondary antibodies used were AlexaFluor 680 goat anti-rabbit (Life Technologies) and IRDye 800 goat anti-rat (Rockland). Reactive bands were visualized with an Odyssey infrared fluorescence imaging system (LI-COR).

### Tetrad analysis

A zero-growth mating procedure was used for sporulation [30]. Individual haploid strains were patched from frozen stocks onto solid YPD and grown overnight. Toothpick scrapings of strains to be mated were then suspended together in 100 μl YPD and 20 μl were immediately spotted back onto solid YPD. Mating was allowed to proceed for 4 h, and the cells were then patched onto SPM. After 2 days, asci were gently digested with Zymolyase 20T (ImmunO, MP Biomedicals) in 1 M sorbitol (Fisher) and tetrads were dissected on solid YPD using a Nikon Eclipse E400 microscope equipped with a dissecting stage. Cells were then allowed to grow for 3 days, upon which viability was assessed, and then replica plated to SC media lacking individual supplements for marker identification. Note that colony color on YPD was used to determine status at the *ADE2* locus: white = *ADE2*^+^, red = *ade2::hisG*. Tetrads exhibiting aberrant segregation at a single marker (five for *HHO1*^+^ and four for *hho1Δ*) were included for spore viability calculations but excluded from tetrad analysis. A single tetrad with aberrant segregation at two loci (*hho1Δ*) was considered false and excluded from all analyses. Genetic map distance [31], including standard error (SE), and crossover interference for individual intervals [32] were calculated using *Stahl Lab Online Tools* (http://www.molbio.uoregon.edu/~fstahl/). Crossover interference between neighboring intervals was analyzed by the coefficient of coincidence, as previously described [33]. P values for crossover interference were determined using statistics calculators available at *VasserStats* (http://www.vassarstats.net). For individual intervals, a Chi square test (one degree of freedom) was used [32]; for adjacent intervals, a binomial probabilities test (two-tailed, normal distribution) was used (see [23, 34]).

## Results and discussion

**Figure 1:**
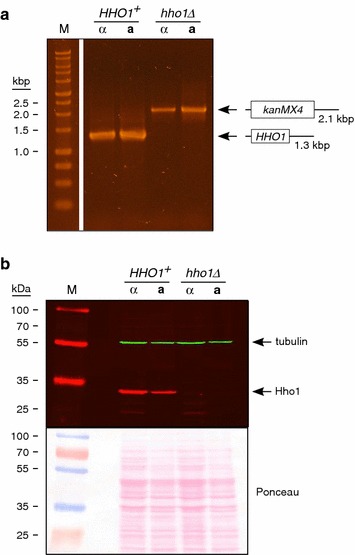
Confirmation of *HHO1* deletion. Four haploid strains used in this study, with indicated *HHO1* status and mating types (*α* or *a*), were analyzed for: **a** the *HHO1* gene by PCR analysis, and **b** Hho1 protein by western blotting. In **a** the positions and expected sizes of PCR products, containing either the *HHO1* open reading frame or the *kanMX4* module (*boxes*), are shown. A DNA ladder (M; Promega) was used for DNA size estimation (kilobase pairs, kbp). In **b** Hho1 (*red*) and tubulin (*green*) staining by western blotting is shown in the *upper panel*, and general protein staining with Ponceau S is shown in the *lower panel*. Pre-stained markers (M; Thermo Scientific) were used for molecular weight estimation (kilodaltons, kDa). A subset of these markers was detected as *red bands* in western blotting. Predicted molecular weights of Hho1 and tubulin are 28 and 50 kDa, respectively (see *Saccharomyces Genome Database* at http://www.yeastgenome.org).

**Figure 2:**
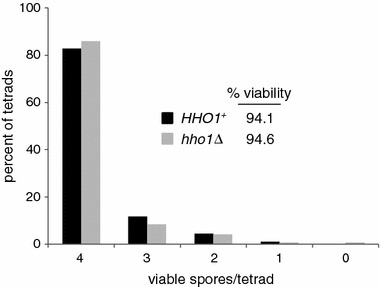
Spore viability profiles in the presence and absence of Hho1. Colony formation after tetrad dissection was used as a metric for spore viability. The percent of tetrads containing the indicated number of viable spores is shown, along with the overall spore viability. The total number of tetrads analyzed was 308 for *HHO1* ^+^ and 307 for *hho1Δ.*

To determine whether Hho1 functions in meiotic recombination, a strain background was used in which several intervals on chromosome XV can be analyzed [23]. Two cell types were compared: those that contained wild type Hho1 (*HHO1*^+^) and those in which Hho1 was absent (*hho1Δ*) (see Figure 1). To specifically examine meiotic recombination and preclude the possibility of detecting mitotic recombination events, individual haploids were mated and then sporulated before significant mitotic growth could occur. Spore viability was determined based on germination. As shown in Figure 2, the spore viability percentages for the two cell types were nearly identical, in agreement with previous studies [16, 20]. The distributions of tetrad types with regard to viable spores, which can provide evidence of an affected process such as recombination (see [23]), were also nearly identical. A recent report has indicated, however, that sporulation efficiency is reduced to some extent in the absence of Hho1 [20]. Therefore, Hho1 is required for optimal sporulation, but for the cells that do complete sporulation and form mature asci, the absence of Hho1 does not appear to be detrimental.

To examine recombination, genetic map distances in four intervals were determined based on the number of parental ditype (PD), tetratype (TT) and non-parental ditype (NPD) tetrads [31]. Results are shown in Table 1. For each interval, the genetic map distance was similar for *HHO1*^+^ and *hho1Δ* cells. There appeared to be a slight difference in the *LYS2*-*ADE2* interval based on the fact that the genetic map distance ± SE of *HHO1*^+^ did not overlap with that of *hho1Δ*. However, the SE of the difference between the two genetic map distances was less than two times the absolute value of this difference, indicating that the genetic map distances were not significantly different (see *Stahl Lab Online Tools* at http://www.molbio.uoregon.edu/~fstahl/). The sums of the genetic map distances of the four intervals gave markedly similar values of 117.80 and 115.00 cM for *HHO1*^+^ and *hho1Δ*, respectively.

**Table 1:** Genetic map distances in *HHO1* ^+^ and *hho1Δ* cells

Interval	Strain	N	PD	TT	NPD	cM ± SE
*URA3*-*LEU2*	*HHO1* ^+^	250	121	126	3	28.80 ± 2.46
	*hho1Δ*	260	126	131	3	28.65 ± 2.38
*LEU2*-*LYS2*	*HHO1* ^+^	250	129	119	2	26.20 ± 2.21
	*hho1Δ*	260	131	128	1	25.77 ± 1.87
*LYS2*-*ADE2*	*HHO1* ^+^	250	169	79	2	18.20 ± 2.17
	*hho1Δ*	260	192	67	1	14.04 ± 1.75
*ADE2*-*HIS3*	*HHO1* ^+^	250	72	169	9	44.60 ± 3.43
	*hho1Δ*	260	78	170	12	46.54 ± 3.73

Genetic map distance in centimorgans (cM) was determined from the number of parental ditype (PD), tetratype (TT), and non-parental ditype (NPD) tetrads. N is the total number of tetrads, and SE is the standard error.

Crossovers are not randomly distributed in most eukaryotes, and budding yeast is no exception. The phenomenon by which double crossovers in a particular region occur less frequently than would be predicted by single crossover incidence is called crossover interference (see [35]). It is thought that this mechanism is important for providing a suitable crossover distribution to help ensure proper chromosome segregation during the first meiotic division. Individual intervals were compared for the frequency of observed NPD tetrads, which arise through double crossovers involving all four chromatids, with the frequency of NPD tetrads expected in the absence of crossover interference [32]. For both *HHO1*^+^ and *hho1Δ* cells, interference was observed in three intervals interrogated (see Table 2), while the *LYS2*-*ADE2* interval was omitted because so few NPD tetrads were expected. Crossover interference was also measured through determination of the coefficient of coincidence, which is based on the frequency of coincident crossover events (indicated by TT plus NPD tetrads) in adjacent intervals [33]. As can be seen in Table 3, *HHO1*^+^ and *hho1Δ* cells showed the same patterns of interference, although the P value for *LYS2*-*ADE2*-*HIS3* was considerably higher for *hho1Δ* than for *HHO1*^+^ cells. It is noted that this general pattern of interference for these regions has been reported in wild type cells of this background [34].

**Table 2:** Crossover interference analysis by NPD ratio in *HHO1* ^+^ and *hho1Δ* cells

Interval	Strain	NPD Ratio (NPD_o_/NPD_e_)	P	I
*URA3*-*LEU2*	*HHO1* ^+^	0.31 (3/9.7)	0.0104	Yes
	*hho1Δ*	0.30 (3/10.0)	0.0082	Yes
*LEU2*-*LYS2*	*HHO1* ^+^	0.24 (2/8.3)	0.0100	Yes
	*hho1Δ*	0.11 (1/9.0)	0.0017	Yes
*ADE2*-*HIS3*	*HHO1* ^+^	0.43 (9/20.7)	0.0005	Yes
	*hho1Δ*	0.56 (12/21.4)	0.0062	Yes

NPD events observed (NPD_o_) and those expected in the absence of crossover interference (NPD_e_) were compared. P < 0.05, based on a Chi square test, is considered statistically significant evidence of crossover interference (I).

**Table 3:** Crossover interference analysis by coefficient of coincidence (COC) in *HHO1* ^+^ and *hho1Δ* cells

Adjacent Intervals	Strain	COC (DCO_o_/DCO_e_)	P	I
*URA3*-*LEU2*-*LYS2*	*HHO1* ^+^	0.70 (44/62.4)	0.0088	Yes
	*hho1Δ*	0.72 (48/66.5)	0.0105	Yes
*LEU2*-*LYS2*-*ADE2*	*HHO1* ^+^	0.51 (20/39.2)	0.0012	Yes
	*hho1Δ*	0.56 (19/33.7)	0.0085	Yes
*LYS2*-*ADE2*-*HIS3*	*HHO1* ^+^	0.80 (46/57.7)	0.0930	No
	*hho1Δ*	0.90 (43/47.6)	0.5093	No

The number of tetrads exhibiting crossover events (TT or NPD) in both of the indicated adjacent intervals (DCO_o_) was compared with the number expected in the absence of crossover interference (DCO_e_). P < 0.05, based on a binomial probabilities test, is considered statistically significant evidence of crossover interference (I).

As suggested previously [20], the decrease in Hho1 observed early in meiosis might be required given that Hho1 inhibits homologous recombination in vegetative cells and, therefore, could be counterproductive during the meiotic recombination phase. Nonetheless, a basal level of Hho1 is still present during this window. The results presented here indicate that complete absence of Hho1 had no obvious impact on meiotic recombination as determined by crossover frequency. Furthermore, apparently normal crossover distribution was maintained in the absence of Hho1. Therefore, a threshold level of Hho1 does not appear to be necessary to restrain reciprocal recombination during meiosis. While overexpression of Hho1 during meiosis could be instructive with regard to the importance of Hho1 depletion for proper recombination, the post-transcriptional mechanism that leads to Hho1 depletion during meiosis (see [20]) may undermine the feasibility of this approach through simple up-regulated transcription. The data presented here also confirm that, despite re-accumulation of Hho1 when spores mature [20], Hho1 is unlikely to be important for proper germination. Thus, Hho1 appears to have minor functional significance during sporulation, at least under laboratory conditions.
